# Environmental, genetic and viral risk factors of nasopharyngeal carcinoma in North Africa

**Published:** 2011-01-10

**Authors:** Nadia Laantri, Marilys Corbex, R’kia Dardari, Abdellatif Benider, Brahim El Gueddari, Meriem Khyatti

**Affiliations:** 1 Institut Pasteur du Maroc, Casablanca, Morocco; 2 WHO Regional Office for the Eastern Mediterranean Region, Cairo, Egypt; 3 Saint Justine Hospital, Montreal, Canada; 4 Oncology Center, CHU Averroes, Casablanca, Morocco; 5 National Institute of Oncology, CHU Avicenne Rabat, Morocco

## 

NPC (Nasopharyngeal carcinoma) is a tumour that arises in the epithelium surface of the posterior nasopharynx and shows a peculiar geographic and ethnic distribution. Intermediate incidence (8-12 cases/100,000/year) was reported in the North African population (7-10% of all cancers among men), where NPC is also the commonest tumour of the ear, nose and throat region.

In Asia, the majority of the cases occur in the fifth and sixth decades of life. In contrast, in North Africa the age distribution is bimodal with a minor peak in people aged between 10 and 25 years old. This juvenile form accounts for approximately 20% of the patients and has specific clinical and biological features. Geographical correlation was also observed between percentages of young cases and consanguinity rates over endemic and non-endemic populations, which may therefore explain the shape of the age curve in North Africa.

Regardless of its incidence and geographic distribution, NPC results from the contribution of environmental, genetic and viral factors. These factors might however combine differently for Asian and North African patients and also among North African patients of the two age groups.

## Environmental factors

The increased risk of NPC was associated with the consumption of rancid butter and rancid sheep fat. Butyric acid contained in these products is considered as a potential Epstein-Barr virus (EBV) activator and a possible causative substance for NPC. In addition, Marijuana smoking was associated significantly to high NPC risk independently of cigarette smoking which suggests dissimilar carcinogenic mechanisms between cannabis and tobacco.

## Genetic factors

Genetic traits play a significant role in the development of NPC. Specific human leukocyte antigen (HLA) haplotypes have been reported to be associated with high risk for NPC, namely HLA-B13 in Tunisians, HLA-A3, B5 and B15 in Algerians and HLA-B18 allele in Moroccans population. In contrast, HLA-Aw33, -B14 and A9 were associated to low risk of NPC in Tunisians, Algerians and Moroccans, respectively.

## Viral factors

EBV is etiologically associated with NPC, and the distribution of Latent Membrane Protein (LMP)-1 variants of EBV in NPC tumors co-segregate with geographic regions. Moroccan NPC patients showed a high prevalence of the 30bp deletion variant of LMP-1; the del-LMP-1 variant share identical amino acid substitutions with the Med variant. Evidence indicates that NPC-derived LMP-1 variants carrying 30bp deletion and specific mutations in the 3C-terminal region confer high oncogenic potential and a weak immunogenicity.

However, although it is widely accepted that EBV is etiologically associated with NPC; it is proven that other co-factors might be involved in the carcinogenesis process, such as HPV (Human papilloma virus) factors. Our results report that 34% of EBV positive Moroccan NPC biopsies harbour HPVs.

